# Combined cortisol and melatonin measurements with detailed parameter analysis can assess the circadian rhythms in bipolar disorder patients

**DOI:** 10.1002/brb3.2186

**Published:** 2021-06-06

**Authors:** Liang Fang, Quanmei Yu, Fanfan Yin, Jiakuai Yu, Yunfei Zhang, Yu Zhang, Daomin Zhu, Ximing Qin

**Affiliations:** ^1^ Department of Psychiatry Affiliated Psychological Hospital of Anhui Medical University Hefei China; ^2^ Department of Sleep Medicine Anhui Mental Health Center Hefei China; ^3^ Institutes of Physical Science and Information Technology Anhui University Hefei China; ^4^ Department of Sleep Medicine Hefei Fourth People's Hospital Hefei China

**Keywords:** biomarker, bipolar disorder, circadian clock, cortisol, gene expression, melatonin

## Abstract

**Objectives:**

Bipolar disorder (BD) is a common chronic mental illness. The circadian clock disorder shows a significant correlation with the pathogenesis, phenotype and recurrence of BD. We aim to evaluate non‐invasive methods that can comprehensively assess the circadian rhythmicity in BD patients.

**Methods:**

We non‐invasively collected salivary samples and oral epithelial cells from recruited subjects. Then the levels of cortisol and melatonin in saliva were measured and the circadian clock gene expressions (*PER2* and *BMAL1*) of epithelial cells were analyzed. Due to the disease characteristics of the manic patients who were difficult to cooperate with the protocol, only one patient at manic episode was recruited. Besides, 11 patients at the depressive episode, 15 healthy controls and four patients at recovery stage were recruited.

**Results:**

Our results exhibited that the peak phase of cortisol level mainly manifested around 8:00 a.m., and the maximal melatonin level reached around 5:00 a.m. The phase of cortisol in patients with depression did not change significantly, but the level of cortisol decreased significantly, while the phase of melatonin level moved forward about 2.5 hr. Furthermore, the levels and phases of cortisol and melatonin in recovery patients tended to be similar to those of healthy controls.

**Conclusions:**

With detailed parameter analysis, the combined detection of melatonin and cortisol can better judge the biological clock disorder of bipolar patients. The circadian rhythms of patients at the recovery stage tend to be normal. The clock gene expression examination needs strict quality control and more investigations before being applied to assess human circadian rhythms.

## Graphical abstract

We comprehensively assess human circadian clocks by combined measurements of cortisol and melatonin. Our detailed parameter analysis of both cortisol and melatonin revealed that a significant phase advance in the bipolar patient at the depressive episode. With strict quality control, we conclude that using gene expression of oral epithelial cell to assess the circadian clock needs cautions.

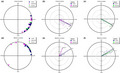

## 1 INTRODUCTION

Bipolar disorder (BD in the following text) is one of the major mental disorders, which manifests in repeated manic and depressive behaviors. At present, about 1%–4% of the world's population suffers from BD disease that often has an evident appearance from adolescence to early adulthood (Fava et al. 2010; Merikangas et al., 2007). BD is a complex disease caused by heredity and environmental susceptibility factors (Lichtenstein et al., 2009). Among these factors, the abnormal circadian rhythm showed a significant correlation with the pathogenesis, phenotype, and recurrence of the disorder. The internal circadian clock controls the daily physiological activities and energy metabolism of the organism from single‐cell level to multiple tissue levels (Balsalobre et al., 1998; Giebultowicz, 2004). Some evidence show that the onset of bipolar disorder is related to the mutation of clock‐related genes. For example, single nucleotide polymorphisms (SNPs) of *NR1D*1/2, *ROR*, and *CLOCK* genes were reported to show relations to the onset of BD (Benedetti et al. 2003; Milhiet et al., 2014; Milhiet et al., 2011). Therefore, the pathogenesis of BD may be closely related to the abnormal biological clock system of the patients.

Light intensity, temperature changes, and other environmental factors could synchronize the body's biological clock, which is called entrainment (Hastings et al., 2003; Pittendrigh, 1960). In these environmental cues, the light/dark cycle is the strongest cue to entrain the clock (Turek et al., 1996). The suprachiasmatic nucleus (SCN) located in the hypothalamus is the pacemaker of the biological clock and the master of the biological clock in mammals that is directly entrained by the light/dark cycle (Hastings et al., 2003). The peripheral circadian clocks exist in various tissues and organs of the body, such as liver, kidney, skin, and so on, which are regulated and coordinated by SCN. Manic symptoms can circulate periodically or even seasonally, which supports that its pathological state has a relationship with environmental cues. In addition, normal sleep‐wake cycle and social timing factors are often essential for emotional stability. When these rhythms are destroyed, a manic episode will be triggered (Saeed et al., 1998). More and more evidence show that the disorder of circadian rhythm is closely related to the pathogenesis and manifestation of BD (Boivin, 2000; Lai et al., 2015; Nováková et al., 2015). Some other research evidence show that the circadian clock of BD patients is out of sync with the external environment, indicated by measuring the blood cortisol or melatonin levels from BD patients (Cervantes et al., 2001; Kennedy et al., 1996; Yang et al., 2009). Thus, one can conclude that there are associations between disturbed circadian rhythms and bipolar disorders during both depression and mania episodes.

Importantly, some exciting preliminary evidence suggests that interventions in light‐dark cycles and sleep‐wake rhythms could potentially enhance the treatments of BD diseases (Frank et al., 2005). Thus, it is critical to assess the circadian rhythm of the BD patients, in order to examine the pathophysiology of the disease. In general, repeated blood samplings are carried out to measure the circulating hormones (such as prolactin, corticotropin, cortisol, thyrotropin, and melatonin) to assess the circadian rhythm (Claustrat et al., 1984; Klerman et al., 2002; Linkowski et al., 1987; Cauter et al., 1996). In some cases, polysomnography studies and sleep logs are recorded to measure the rhythm (Roenneberg et al., 2007). However, repeated blood sampling is not feasible to carry out on patients with severe depression or mania since it is invasive and patients may not be able to tolerate such a procedure. Questionnaire studies are too subjective to reflect the endogenous status of the patients. Recently, single or multiple sample estimations of internal circadian rhythms were established by analysis of blood metabolites or gene expression, but these methods are still at a very early stage and are not adapted for clinical usage (Ueda et al., 2004). Therefore, non‐invasive measurements are required to assess the circadian rhythm of the patients.

In the past decades, non‐invasive assays have been applied to measure the circadian rhythms of human beings, such as measuring the saliva cortisol (Kirschbaum & Hellhammer, 1994), saliva melatonin (Zhou et al., 2003), and gene expression patterns from buccal cells (Cajochen et al., 2006; Cho et al., 2016; Nováková et al., 2015). Dysregulated hormone levels or disordered circadian rhythms in BD patients have been suggested by these studies, but inconsistent results have been reported. Some research has demonstrated that saliva melatonin levels were found to be decreased in patients with BD and the dim light melatonin onset (DLMO) was found to be later than controls (Parry & Newton, 2001; Robillard et al., 2013). Others reported increased cortisol levels while some others did not find such differences (Cervantes et al., 2001; Deshauer et al., 2006; Havermans et al., 2011). Others evidence shows phase advance in the BD patients at mania but not at depression episode (Nováková et al., 2015). Moreover, Huang et al. reported lower cortisol expression and a blunted cortisol awakening response (CAR) in depressive BD patients (Huang et al., 2017). The exact profiles of these circadian biomarkers in the BD patients which could be important for the treatments of BD diseases remain elusive.

The aim of this study was to evaluate the valid examinations that can measure the circadian rhythm from BD patients using non‐invasive sample collections. The main subjects we recruited for this study were BD patients at the depression episode and a few of them were at the recovery phase. Then we studied the daily pattern of the potential circadian rhythms of cortisol, melatonin, and clock gene expression within the recruited BD patients. We found that the rhythms of melatonin and cortisol are robust in control groups, depressive BD patients, and BD patients at recovery phase. These rhythms showed different patterns in depressive BD patients from the healthy control group, while they displayed similar profiles with those of healthy controls and recovery BD patients. However, the gene expression pattern in buccal mucosa cells cannot be validated since the quality of extracted RNA is low, which needs further examination. Our data suggest that the combination of measuring cortisol and melatonin could be a solid and comprehensive examination to distinguish depressive BD patients from healthy controls while measuring the gene expression level should be a very cautious way to undertake.

## 2 MATERIALS AND METHODS

### 2.1 Participants

The patients with BD were recruited from inpatients at the Department of Sleep Disorders, Psychological Hospital Affiliated to Anhui Medical University. From March 2019 to February 2020, the study included 11 patients with bipolar disorder at depressive stages who were hospitalized in the Anhui Medical University's affiliated psychological hospital (three males and eight females with the average HAMD scores 30.83 ± 3.971, indicating that they were at moderate to severe depressive episodes), one BD patient at mania episode, and four BD patients at recovery stage (three males and and female), who were recruited from outpatient clinics through telephone calls. At the same time, 15 healthy controls (six males and nine females) were recruited from the community. The ages of the participants were 30.81 ± 12.913 (mean ± *SD*) years old for patients and 25.53 ± 8.903 years old for healthy controls, respectively. There were no significant differences with respect to characteristics including age and sex among groups (Table 1). Detailed group information and corresponding numbering of each recruit are listed (Table 2). [Correction added on 17 June 2021, after first online publication: The word "one" has been deleted.]

**TABLE 1:** Demographic data of the recruited subjects

	Patients with BD	Healthy controls	*p* value
Number of subjects	16	15	–
Age (mean ± *SD*), years	30.81 ± 12.913	25.53 ± 8.903	.192
Sex (M/F), *N*	6/10	6/9	.589
Education (mean ± *SD*), years	14.56 ± 2.308	14.87 ± 2.386	.721
Age of onset (mean ± *SD*), years	26.25 ± 10.969	N/A	–
Total number of mood episodes (mean ± *SD*)	5.94 ± 8.22	N/A	–
Total number of psychiatric hospitalizations (mean ± *SD*)	1.50 ± 0.817	N/A	–

**TABLE 2:** Numbering system to recruited subjects

Controls	LA1,LA2,LA3,LA4,LA5,LA6,LA7,LA8,LA9,LA10,LA11,LA12,LA13,LA14,LA15
Depression	LB1,LB2,LB4,LB5,LB6,LB7,LB8,LB12,LB14,LB15,LB16
Mania	LB3
Recovery	LB9,LB10,LB11,LB13

Bipolar disorder patients all meet the diagnosis of BD in the International Classification of Diseases and Related Health Problems (Tenth Edition; ICD‐10) (Prigerson et al., 2009). Patients with BD in various clinical stages were recruited for the study. Inclusion criteria of the participants were: 1) Age 16–65 years old; 2) Diagnosis of BD according to ICD10 criteria; 3) Junior high school and above education level and 4) Understand the research content and sign the informed consent. Patients or controls with intellectual disability, organic brain injury, or other major psychiatric disorders were excluded from the present study, including those who have received electroconvulsive therapy or transcranial magnetic stimulation within 3 months and are unable to take medication as prescribed. All participants underwent screening to exclude past or present major medical disorders such as diabetes, thyroid disease, hypertension, heart disease, narrow‐angle glaucoma, epilepsy, dementia, and other neurological diseases and history of pregnancy or breastfeeding women, or those planning to become pregnant. At the same time, our exclusion criteria include no smoking, no caffeinated drinking, and no medications of hydrocortisone or prednisolone, etc. Other medications are allowed during the research. Clinical symptoms were evaluated using the Yang's Mania Rating Scale (YMRS) (Young et al., 1978), the 24‐item Hamilton Depression Scale (HAMD) (Hamilton, 1980), and the Hamilton Anxiety Scale (HAMA).

Participants were informed of the purpose and procedures of the study. After fully explaining and fully understanding the study, all participants provided informed written consent before recruitment. The research plan was approved by the Institutional Review Committee of Psychological Hospital affiliated to Anhui Medical University.

### 2.2 Protocol

**FIGURE 1:**
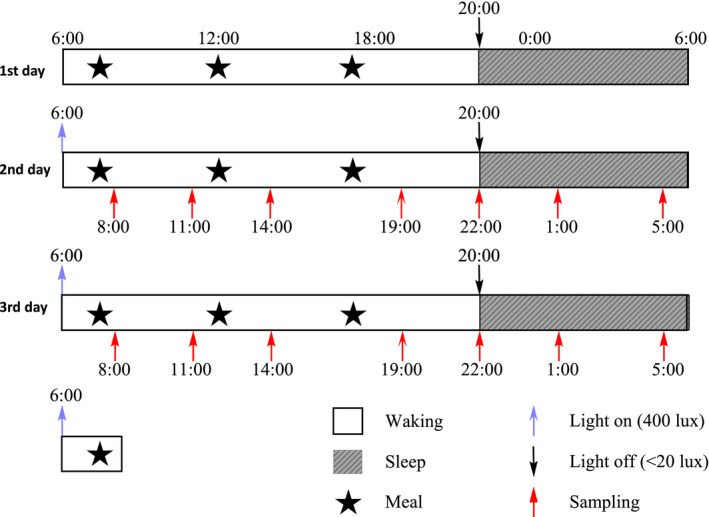
Schematic of the protocol design. The figure illustrates the protocol used in this study. We have two isolated sleep monitoring laboratories which have its light on at 06:00 (indicated by the blue arrow) and light off at 20:00 (indicated by the dark arrow). Recruited BD patients and healthy controls who are maintaining a 22:00–06:00 sleep schedule. In the laboratory, subjects can maintain their normal activity without using electronic books or smart telephones during waking time. We believe that this situation is as close to the real situation as possible. The subjects took 1 day to get adapted to the laboratory environment, and saliva samples were samples at indicated time points (08:00, 11:00, 14:00, 19:00, 22:00, 01:00, and 05:00) in the two consecutive days. Since most subjects cannot tolerate the procedure of buccal mucosa sampling, this process takes 1 day at indicated time points. Mealtime (normal meal with low‐calorie intake) was controlled during the whole procedure. Light levels were maintained at about 400 lux during the light on period and less than 20 lux during the light‐off period

Patients with bipolar disorder were recruited and diagnosed by Psychological Hospital affiliated to Anhui Medical University during periods of depression or manic episodes. The normal control group had excluded the history of personal or family psychosis. The subjects had regular sleep/awakening schedules during the week before sampling. As indicated in Figure 1, the sleep habits of hospitalized subjects were controlled by the regular ward routine, that is, they went to bed at 22:00 and waked up at 06:00 a.m. Light exposure was also controlled by the ward routine, that is, the lights were turned off from 22:00 to 06:00 (Light intensity does not exceed 20 lux), and turned on from 06:00 to 22:00(light intensity is about 400 lux). All subjects avoid prolonged access to natural daylight, and need to adapt to the ward for one day, and oral mucosal cells were collected on the second and third days. Samples are collected by well‐trained nurses. Subjects are not allowed to drink alcoholic and caffeinated beverages during the sampling period, use gum or brush their teeth frequently. After providing saliva (≥1 ml) directly into Salivettes (Sarstedt AG & Co) which were stored at −40°C until detection, buccal epithelial cells were collected and immediately placed into RNA lysis solution (Sigma‐Aldrich) and stored at −40°C until detection. The nighttime sample collections were performed by well‐trained nurses without additional light exposure (Light intensity does not exceed 20 lux), under light as dim as possible, but these collections did require brief awakenings, and it is known that patients with acute psychiatric illnesses usually have disturbed sleep. After a few sampling, the recruited subjects could not tolerate the procedure of collecting buccal epithelial cells for two consecutive days. Thus, the saliva samples were collected for consecutive 2 days, but the buccal cells were collected for 1 day.

### 2.3 Measurement of salivary cortisol

Salivary cortisol was assayed using Salivary Cortisol ELISA (DRG International, Inc), and used in strict accordance with the product instructions. The analytical sensitivity is 0.09 ng/ml. The intra‐assay coefficient of variation is 6.1% for 3.08 ng/ml samples and 2.6% for 20.14 ng/ml samples. The inter‐assay coefficient of variation is 13.6% for 0.64 ng/ml samples and 4.3% for samples of 19.78 ng/ml. The salivary cortisol level at each time point is expressed in ng/ml and expressed as the mean ± *SEM*.

### 2.4 Measurement of salivary melatonin

Salivary melatonin was assayed using Melatonin direct Saliva ELISA (IBL International GmbH) and used strictly in accordance with product instructions. The analytical sensitivity was 0.3 pg/ml. The intra‐assay coefficient of variation is 10.8% for 1.7 ± 0.2 pg/ml samples and 8.7% for 33.2 ± 2.9 pg/ml samples. The inter‐assay coefficient of variation is 12.7% for 2.1 ± 0.3 pg/ml samples and 13.0% for the sample of 14.7 ± 1.9 pg/ml. The melatonin levels at each time point are expressed in pg/ml, and expressed as mean ± *SEM*.

### 2.5 Measurement of circadian gene expression

The expression levels of circadian rhythm‐related genes *PER2* and *BMAL1* were determined by reverse transcription‐quantitative polymerase chain reaction (RT‐qPCR) and the BMAL1/*PER2* ratio at each sampling time point was used to measure circadian rhythm. Total RNA was isolated using RNAprep pure Micro Kit (Tiangen) from buccal epithelial cells. The whole RNA sample was reverse‐transcribed using the Sensiscript Reverse Transcription Kit (Tiangen). Then, cDNA was subjected to the quantitative real‐time polymerase chain reaction using an Applied Biosystems real‐time PCR systems (Thermo Fisher Scientific). The primers used in this experiment are as follows; *PER2* (NM_022817): forward 5′‐GCAAAATCTGAACACAACCC‐3′, reverse 5′‐CTTTGTGTGTGTCCACTTTC‐3′; *BMAL1* (NM_001030272): forward 5′‐ACATGCAACGCAATGTCCAG‐3′, reverse 5′‐TCTGTGTATGGATTGGTGGC‐3′;*RPLPO* (NM_001002): forward 5′‐ACGGGTACAAACGAGTCCTG‐3′, reverse 5′‐GCCTTGACCTTTTCAGCAAG‐3′.

### 2.6 Statistical analysis

**FIGURE 2:**
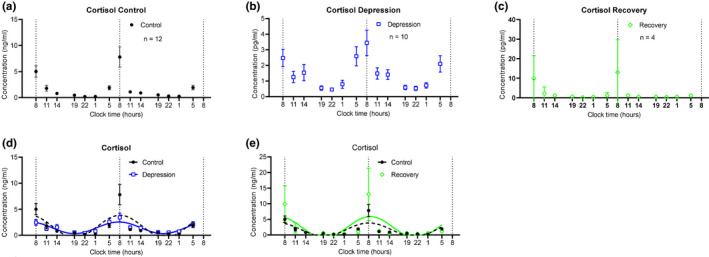
Two consecutive day profile of cortisol levels in collected saliva samples from recruited subjects. (a) Averaged cortisol measurement from healthy controls. Saliva samples were taken at conditions as illustrated in Figure 1. (n =12, mean±SEM ) (b) Averaged cortisol measurement from BD patients at depression episode. (n =10, mean±SEM ) (c) Averaged cortisol measurement from BD patients at recovery phase. (n =4, mean±SEM ) (d) Cosine curves were used to fit the measured cortisol values from both healthy controls and depressive BD patients. The dashed line presents the fitted curve of healthy controls, and the solid blue line presents the fitted curve of depressive BD patients. (e) Cosine curves were used to fit the measured cortisol values from both healthy controls and recovery BD patients. The solid green line presents the fitted curve of recovery BD patients. Note that these values are two consecutive days, which show robust rhythms. Also note that the scales of Y ‐axis of panels A, B, C are different [Correction added on 17 June 2021, after first online publication: The figure has been updated.]

**FIGURE 3:**
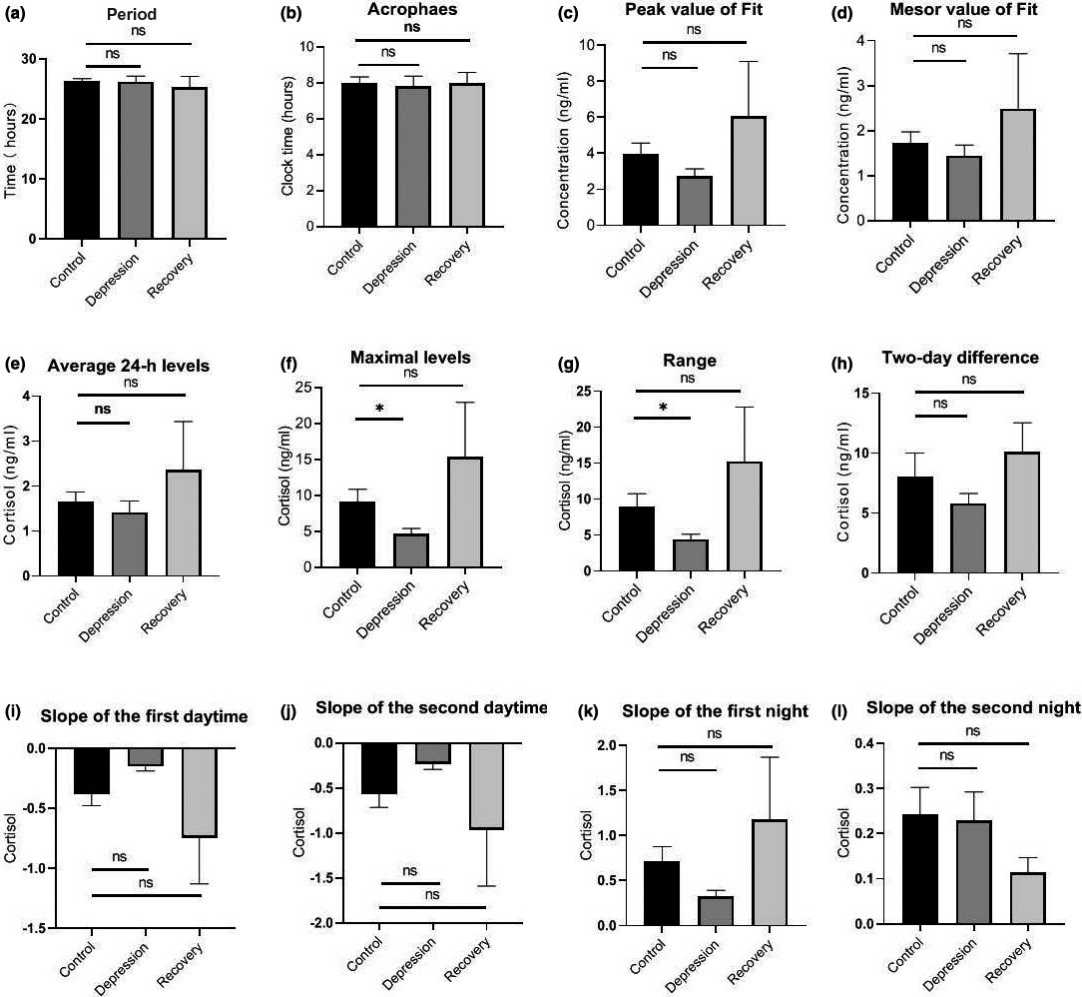
Analysis of various parameters of saliva cortisol secretion in two consecutive days from recruited subjects. (a) Period of the cortisol rhythm, calculated by the fitted cosine curve in Figure 2. (b) The acrophases of saliva cortisol in healthy controls, depressive BD patients, and recovery BD patients, based on the fitted curves in Figure 2. (c) The peak values of saliva cortisol in the fitted curves for each group. (d) The mesor values of saliva cortisol in the fitted curves for each group. (e) Average cortisol levels for the two consecutive days. (f) The maximum value of cortisol for each group at the clock time 08:00 on the second day. (g) The difference between the maximum level and lowest level of cortisol for each group. (h) Two‐day differences of cortisol for each group, calculated by the average absolute values of the difference between two matching time points in the first and second days. (i) Slope of the first daytime is defined as the slope by fitting linearly to the cortisol level at time points 08:00, 11:00, and 14:00. (j) Slope of the second daytime, calculated using the cortisol values on the second day. (k) Slope of the first night is defined as the slope by fitting linearly to the cortisol level at time points 01:00, 05:00, and 08:00. (l) Slope of the second night, calculated using the cortisol values on the second day. ns, non‐significant; *, p value <.05 [Correction added on 17 June 2021, after first online publication: The figure has been updated.]

The 24‐hr saliva melatonin and cortisol concentration levels of each subject were statistically plotted. According to each person's saliva cortisol and melatonin data, detailed characteristic data were extracted, such as average 24‐hr levels, maximal levels, range, two‐day difference (defined by the accumulation of the difference at the same time point on both days), the slope of the daytime and night during the 2 days. These detailed parameters were plotted in Figures 2,3.

Each person's melatonin data is nonlinearly fitted by the definition of Y = mesor [amplitude * cos (2 [X‐acrophase]/wavelength)], and the wavelength is constant at 24 hr (Nelson et al., 1979). The saliva cortisol data of each person is nonlinearly fitted by the definition formula Y = mesor [amplitude * sin (2 [X–acrophase]/wavelength)], and the wavelength is constant at 24 hr. Using Prism version 8 software (GraphPad) to achieve nonlinear curve fitting, calculate the mesor, amplitude, acrophase of the curve, and coefficient of determination *R* ^2^ (that is, the goodness of fit). Then the circadian rhythm variables of each person's saliva cortisol and melatonin were extracted separately, such as wavelength, acrophase, fitted peak value, and fitted mesor.

Statistic graphs of various characteristic data and circadian rhythm variables of the control group and BD patients were plotted as mean ± *SEM*. Student's *t* test was used to compare the characteristic data and circadian rhythm variables of the control group and BD patients, with a significant *p* < .05. Convert the peak phase of each person into phase angle, calculate and draw the average vector of the peak phase angle and the circular distribution of the peak value of each group. The Watson–Williams *F* test was used to assess differences between groups.

Due to irreversible reasons such as experimental operation (calculated value is out of the range of the standard curve) and sample quality, data at some time points are either dropping or missing. Participants who have missing data at more than four time points are not included in the final statistical analysis. Participants who do not perform statistical analysis include LA11, LA12, LA13, LB15 (cortisol data) and LA11, LA14, LA15, LB8, LB12, LB13,LB16 (melatonin data). There is only one person in the manic phase of bipolar disorder, which is not statistically significant, so statistical analysis is not performed.

## 3 RESULTS

### 3.1 Daily salivary cortisol profiles are similar in healthy controls and BD patients in the depressive episode, but the peak cortisol level of BD patients is lower than healthy controls

The cortisol profiles of all subjects, including healthy controls and recruited BD patients, showed significant circadian rhythms when the cortisol contents in two consecutive days collected saliva were measured. A cosine analysis exhibited significant fits to each individual (Figures S1,S3). Averaged data of the cortisol profiles were demonstrated according to the three groups: healthy controls (control, Figure 2a), BD patients in the depressive episode (depression, Figure 2b), and BD patients in the recovery phase (Recovery, Figure 2c). Also, the cortisol profiles were compared in control versus depression (Figure 2d), and control versus recovery groups (Figure 2e). Next, based on the averaged cortisol values of each group, we calculated various parameters of the salivary cortisol. The periods of the three groups were not different (Figure 3a), nor were the calculated peak cortisol phases (Figure 3b). After fitting the cortisol profile with cosine curves, the peak values and mesor values were extracted and compared in the three groups and showed no differences (Figure 3c,d). Averaged raw measured values were used to calculate the following parameters: Averaged 24‐hr levels (Figure 3e), Maximal levels (at 08:00 clock time, Figure 3f), Range (Figure 3g), and Two‐day difference (Figure 3h). Among them, the maximum value of cortisol in the Depression group is significantly lower than that of the controls (Figure 3f, *p* = .0439, *t* test), and the overall cortisol range is significantly smaller in the depression group (Figure 3g, *p* = .0382, *t* test). The cortisol levels at time points 08:00, 11:00, and 14:00 were linearly fitted to calculate the decline slope, while these at 01:00, 05:00, and 08:00 were fitted to calculate the incline slope. In the two consecutive days, none of the slopes showed significant differences (Figures 3i–l). But the decline slopes (slope of the daytime) had smaller values in the depressive patients than these of healthy controls (Figure 3i,j). [Correction added on 17 June 2021, after first online publication: The figure citation has been updated.]

**FIGURE 4:**
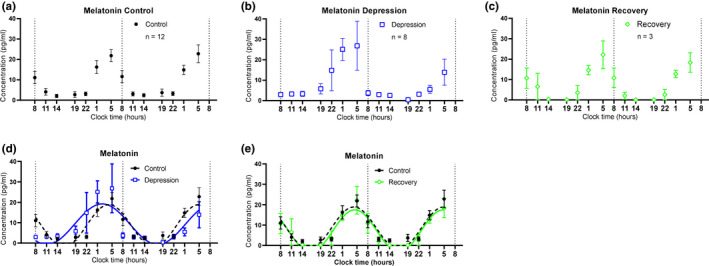
Two consecutive day profile of melatonin levels in collected saliva samples from recruited subjects. (a) Averaged melatonin measurement from healthy controls. Saliva samples were the same as these for the cortisol measurements. (n =12, mean±SEM ) (b) Averaged melatonin measurement from BD patients at depression episode. (n =8, mean±SEM ) (c) Averaged melatonin measurement from BD patients at recovery phase. (n =3, mean±SEM ) (d) Cosine curves were used to fit the measured melatonin values from both healthy controls and depressive BD patients. The dashed line presents the fitted curve of healthy controls, and the solid blue line presents the fitted curve of depressive BD patients. (e) Cosine curves were used to fit the measured melatonin values from both healthy controls and recovery BD patients. The solid green line presents the fitted curve of recovery BD patients. X ‐axis shows the collecting time points at the normal clock time [Correction added on 17 June 2021, after first online publication: The figure has been updated.]

### 3.2 Phases of the daily melatonin profiles in BD patients in the depressive episode are advanced than that in healthy controls

**FIGURE 5:**
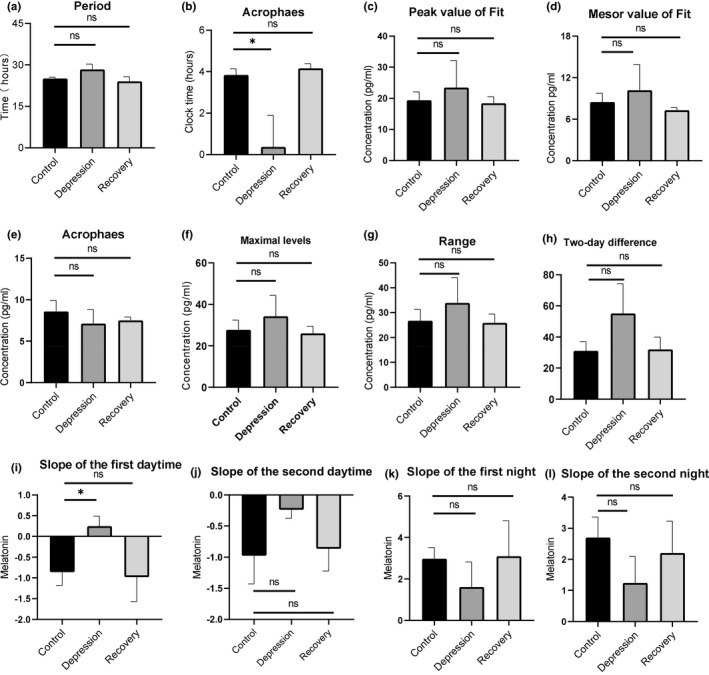
Analysis of various parameters of saliva melatonin secretion in two consecutive days from recruited subjects. (a) Period of the melatonin rhythm, calculated by the fitted cosine curve in Figure 4. The period of the depression group was slightly longer, but not significant. (b) The acrophases of saliva melatonin in healthy controls, depressive BD patients, and recovery BD patients. It was significantly phase‐advanced in the depression group. (c) The peak values of saliva melatonin in the fitted curves for each group. (d) The mesor values of saliva melatonin in the fitted curves for each group. (e) Average melatonin levels for the two consecutive days. (f) The maximum value of melatonin for each group at the clock time 05:00 on the second day. (g) The difference between the maximum level and lowest level of melatonin for each group. (h) Two‐day differences of melatonin for each group, calculated by the average absolute values of the difference between two matching time points in the first and second days. (i) Slope of the first daytime is defined as the slope by fitting linearly to the melatonin level at time points 08:00, 11:00, and 14:00. (j) Slope of the second daytime, calculated using the melatonin values on the second day. (k) Slope of the first night is defined as the slope by fitting linearly to the melatonin level at time points 22:00, 01:00, and 05:00. (l) Slope of the second night, calculated using the melatonin values on the second day. ns, non‐significant; *, *p* value < .05

Similarly, the melatonin profiles of all subjects, including healthy controls and recruited BD patients, showed significant circadian rhythms. The cosine analysis (see M&M) exhibited significant fits to each individual, with a better fitness than that of cortisol profiles (*R* ^2^ values are significantly higher, Figures S4,S6). Averaged data of the melatonin profiles were demonstrated according to the three groups: healthy controls (Control, Figure 4a), BD patients in the depressive episode (Depression, Figure 4b), and BD patients in the recovery phase (Recovery, Figure 4c). Also, the melatonin profiles were compared in control versus depression (Figure 4d), and control versus recovery groups (Figure 4e). Like we did for the cortisols, the various parameters of the salivary melatonin were calculated. The periods of melatonin in the three groups were not different (Figure 5a). However, the peak phases of melatonin in the depression groups were significantly advanced than those in the control groups (Figure 5b, *p* = .0139, *t* test). The other parameters, such as Peak value (Figure 5c), Mesor value (Figure 5d), Average 24‐hr level (Figure 5e), Maximal levels (Figure 5f), Range (Figure 5g), and Two‐day difference (Figure 5h) were not different in three groups. The melatonin levels at time points 08:00, 11:00, and 14:00 were linearly fitted to calculate the decline slope of the daytime, while these at 22:00, 01:00, and 05:00 were fitted to calculate the incline slope during the night. The slope of the first daytime was significantly different between the control and depression groups (Figure 4d & Figure 5i, *p* = .0212, *t* test). The other three parameters of the slopes were not different in three groups (Figures 5j–l).

### 3.3 Both the salivary cortisol and melatonin levels are comparable in BD patients in the recovery phase with these in healthy controls

**FIGURE 6:**
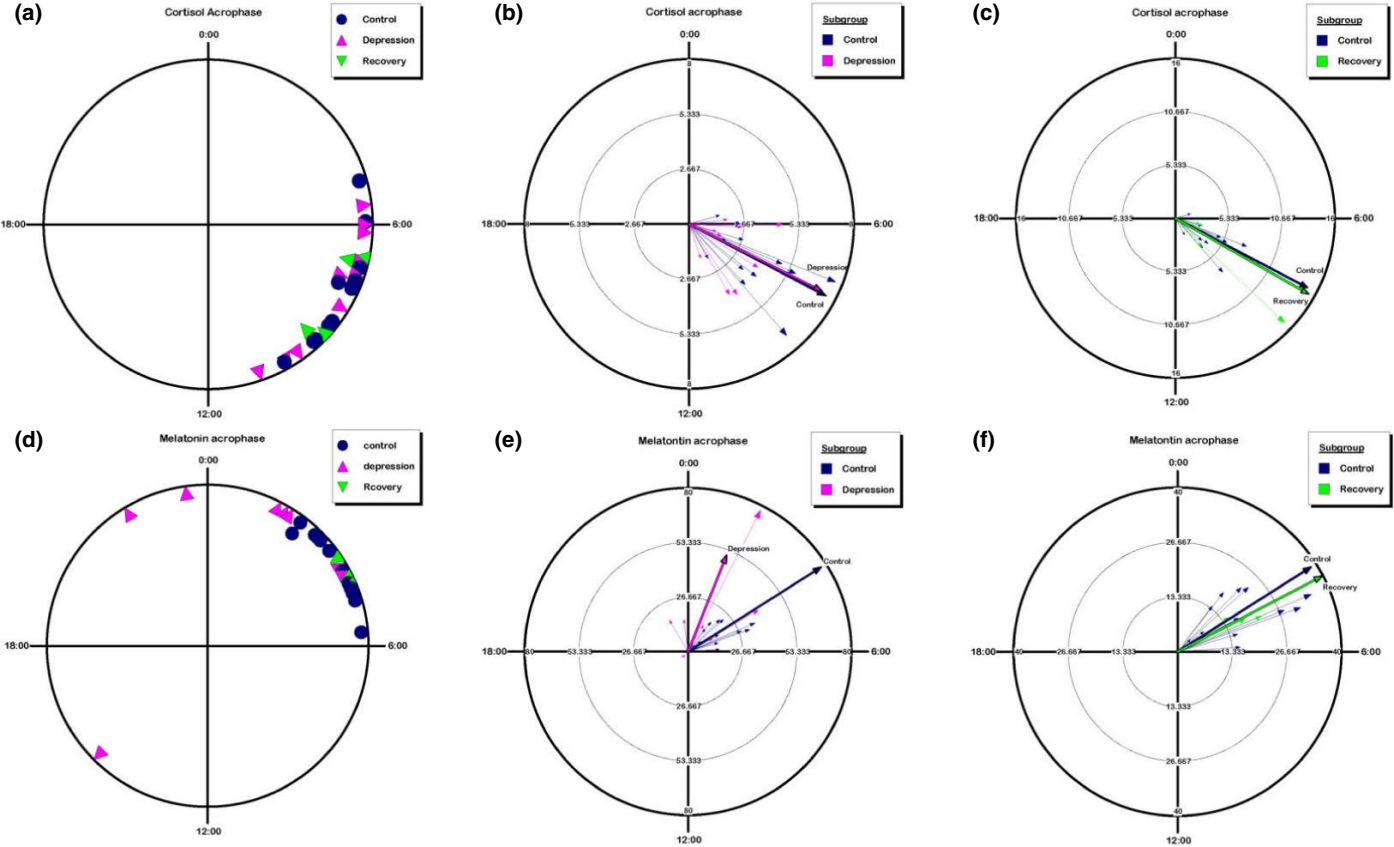
The acrophases of salivary cortisol and melatonin of each individual subject, revealed by Rayleigh plots. (a) Individual acrophase of peak cortisol level, assessed by the fitted cosine curve of the measured cortisol profile. Rayleigh plots indicate a 24 hr clock, with regular clock time 00:00 at the top and 12:00 at the bottom of the circle. Navy circles represent the healthy controls. Fuchsia triangles represent the recruited BD patients at depression episodes. Green triangles represent the recruited BD patients at the recovery phase. (b) For the healthy controls and the BD patients at the depressive episodes, the peak phases of salivary cortisol of individual subjects are presented in the Rayleigh plots. The small arrows indicate the data of each person，arrow points to the responding clock time of each peak phase. The length of the vector represents the strength of the fitted curve of that individual subject. Mean vectors of each group (control versus depression) were also calculated and represented with the labeled color, respectively. The length of the average vector represents the dispersion, and the direction represents the average peak time. (c) The peak phases of salivary cortisol of individual subjects of both control and recovery groups are presented, respectively. Mean vectors were calculated and represented with the labeled color. (d) Individual acrophase of peak melatonin level, assessed by the fitted cosine curve of the measured melatonin profile. (e) The peak phases of salivary melatonin of individual subjects of both control and depression groups are presented, respectively. (f) The peak phases of salivary melatonin of individual subjects of both control and recovery groups are presented, respectively

In order to better present the peak phases of the salivary cortisol and melatonin in recruited subjects, Rayleigh plots were applied to display the calculated acrophases of both hormones. Peak phases of each individual were converted into phase angles relative to the clock time. The cortisol peak phases of individuals in three groups, control/depression/recovery, were plotted (Figure 6a). Mean vectors of circular distributions based on individual measurements were calculated and plotted. Two comparisons, control versus depression (Figure 6b) and control versus recovery (Figure 6c), were analyzed separately. The melatonin peak phases of each individual (Figure 6d) and the comparisons (Figure 6e,f) were carried out using the same analysis method. Watson‐Williams *F* test was applied to evaluate the differences among three groups. According to the analysis, the peak phase of salivary melatonin is advanced in the depression group, compared to healthy controls (Figure 6e, *p* = .058, Watson‐Williams *F* test). This difference was also indicated in the early parameter analysis (Figure 5b). Interestingly but not surprisingly, both the cortisol peak phases and melatonin peak phases are very similar in the recovery groups and control groups (Figure 6c,f). Besides, not only the peak phases of these two hormones but also the other parameters such as the maximal levels of cortisol (Figure 3f), range of cortisol (Figure 3g), slope of the first daytime of melatonin (Figure 5i) all show similar values between the control and recovery groups. [Correction added on 17 June 2021, after first online publication: The figure citation has been updated.]

### 3.4 Special attentions are required when the clock gene expression in buccal mucosa cells was used to assess the circadian rhythms in either healthy controls or BD patients

Based on previous limited buccal mucosa gene expression studies (Cajochen et al., 2006; Cho et al., 2016; Nováková et al., 2015), the patterns of *BMAL1*/*PER2* expression ratios were utilized to assess the gene expression circadian rhythms. We noticed that previous studies have not reported stabilities of the extracted RNA from collected buccal cells (Cajochen et al., 2006; Cho et al., 2016; Nováková et al., 2015). Thus, we evaluated the qualities of extracted RNAs, including control RNAs that were extracted from cultured U2OS cells. According to the cell counting assay, each buccal mucosa cell collection could normally harvest around 100,000 cells (data not presented). Two different amounts of U2OS cells, cells from 10 cm dish and 1 × 10^5^ cells, were collected and RNAs were extracted. Both RNA samples showed high values of RIN and 28S/18S that indicate good qualities of extracted RNA (Table 3). When RNAs were extracted from collected buccal mucosa cells, though total quantities were comparable to these RNAs extracted from 1 × 10^5^ mucosa cells, the qualities did not reach the standard. Both RIN and 28S/18S values were low, indicating the mucosa RNAs were not stable and possibly in highly degraded states (Table 3). Nevertheless, we further tested the *BMAL1*/*PER2* expression patterns of collected one‐day samples. The acrophases of the gene expression patterns were evenly distributed in 1 day, and the amplitudes showed large variances (Figures S7 and S8). Based on our observations, we highly recommend special cares are required for this gene expression method used for the assessment of circadian rhythms in humans.

**TABLE 3:** Stability test of the RNA extracted from different sources

Sample	Concentration (RNA, ng/μl)	Volume (μl)	Total quantity (μg)	RIN value^a^	28S/18S^b^
Buccal mucosa sample‐A	4.82	15	0.0723	2.7	N/A
Buccal mucosa sample‐B	1.61	15	0.0242	1.7	N/A
U2OS large sample‐A	237.12	25	5.928	9.8	3.1
U2OS large sample‐A	216.76	25	5.419	9.8	3
1 × 10^5^ U2OS‐A	8.03	20	0.161	9.7	2.7
1 × 10^5^ U2OS‐B	8.06	20	0.161	10	2.7
1 × 10^5^ U2OS‐C	20.01	20	0.400	9.2	2.4

^a^ RIN value indicates the degradation degree of extracted RNA. Normally RIN value should be higher than seven to be considered as intact RNA.

^b^ 28S/18S is the parameter that indicates the stability of extracted RNA. Intact extracted RNA should has 28S/18S > 0.7.

## 4 DISCUSSION

This study demonstrates that the depression episodes in BD patients are associated with disturbed circadian rhythms through investigating the circadian biomarker cortisol and melatonin non‐invasively. We clearly observed ~2.5 hr phase advances of melatonin rhythms in the BD patients at depressive episodes (Figures 4, 5b, 6b). The peak phase of melatonin in healthy controls reaches to maximal level around 03:50 clock time during sleep time, while that in depressive BD patients reaches to maximum around 01:30 clock time. Besides, we observed that the amplitude of cortisol is significantly damped in the depressive BD patients than that in the healthy controls (Figure 3f,g). Not only because of the difficulty to recruit BD patients at mania episode but also due to the prevalent COVID‐19, we only had one mania patient recruited for this study. Though not statistically representative, this mania patient showed highly advanced acrophase of melatonin (Figure S9). The melatonin phase in this patient was >7 hr advanced compared to the controls. Very interestingly, the amplitude of cortisol level and the phase of melatonin level of BD patients at recovery phase were comparable to these in controls (Figures 3f, 5b, 6c,d), which indicate corrected circadian rhythms may reflect a proper treatment.

Previous studies have suggested that disturbance of circadian rhythms is a possible cause of BD (Duarte Faria et al., 2015; Gonzalez, 2014). Most studies concentrated on measuring only cortisol level or only melatonin level and inconsistent results were reported (Deshauer et al., 2006; Havermans et al., 2011; Parry & Newton, 2001). There has no measurement of circadian rhythms of both cortisol and melatonin through non‐invasive collections with detailed parameter analysis including not only periods, amplitudes, but also the rising and decreasing slopes of each hormone. This study provides detailed parameter analysis to comprehensively assess the rhythmicity and reveals that the decreasing slopes of melatonin during daytime were smaller in depressive BD patients (Figure 5). This detailed parameter analysis provides more information to assess the circadian rhythm of a given subject. For example, averaged 24‐hr levels, maximal levels, ranges, two‐day differences, and slopes of both daytime and night showed no difference between healthy controls and recovery groups (Figures 3,5), indicating an effective therapy results in those patients. Thus, this method may be used to objectively evaluate whether a BD patient is treated to a satisfied level. One potential weakness of this method is that it will be difficult to compare the level of cortisol or melatonin when large numbers of samples are measured, which need internal controls from batch to batch.

Salivary cortisol concentrations can reflect plasma unbound cortisol levels and there was almost no time lag between salivary cortisol and plasma cortisol values (Kirschbaum & Hellhammer, 1989). Similarly, salivary melatonin concentrations show strong correlations with plasma levels, though salivary melatonin is about three times less than that in plasma (Ito et al., 2013). Thus, body circadian rhythms can be assessed based on changes in both hormone levels. Our results show that the maximal level of cortisol peaks around 08:00 clock time in all the recruited subjects, which agrees with most of the cortisol studies in humans. Cortisol is important to maintain body blood pressure, blood sugar, and respond to stress. In the depressive BD patients, the maximal cortisol level was significantly smaller. This observation indicates the adrenal function might not be fully activated in these depressive patients. Our observations that the phases of cortisols are the same in the three groups indicate that the secretion of this hormone might be regulated primarily by the light/dark cycle, rather than the endogenous circadian pacemaker. In contrast, the salivary melatonin level in the depressive BD patients was significantly phase‐advanced than healthy controls. Our results indicated that melatonin can better reflect the endogenous circadian rhythm. Our results showed that an advanced phase in the BD patients at the depressive episodes (Figures 4d,5b), while the patients with other depressive symptoms showed delayed or lengthened circadian rhythm (Robillard et al., 2013). This difference indicates different pathogenesis of BD from other depressions, though they may appear similar depressive symptoms. Taking consideration of different phase angles in BD from other depressions is very interesting, a recent study has classified patients with depressive disorders into two subgroups based on the phase angle between dim light melatonin onset and core body temperature (Robillard et al., 2018). Also, the relative early phase of the melatonin in depressive BD patients may explain more sleepiness in BD patients at the depression episodes. The only one mania patient showed a very large phase advance, compared to controls. Whether this represents shorter circadian cycles in mania BD patients cannot be proven in this study. We will recruit more patients in our future study. Previous studies have reported suppressed melatonin levels in the bipolar patients (Lam et al., 1990), which we did not observe in this study. This difference might be due to different sampling times over 1 day.

Besides cortisol and melatonin, we attempted to measure gene expression levels using canonical circadian genes, *BMAL1* and *PER2*. Unfortunately, our results showed low RIN values and very low 28S/18S values of the RNAs extracted from buccal mucosa cells. RNAs extracted from similar small amount of cultured cells (1 × 10^5^) showed good quality both in RIN and in 28S/18S values. These measurements highly suggest instability or lack of intact in these mucosa extracted RNAs, since RNase and other nuclease are abundantly present in saliva (Kumar et al., 2006). Though previous studies have reported using gene expression profiles to assess the circadian rhythms (Bjarnason et al., 2001; Cho et al., 2016; Jud et al., 2009; Koshy et al., 2019), none of them have done RNA stability tests before using cDNA reversely transcribed from extracted RNAs. Thus, we highly recommend that special attention and more controlled experiments are required to further solidify the circadian rhythm assessment using the gene expression method.

In our measurement protocol, recruited controls and patients are in an environment that simulates their normal living conditions. Our exclusion criteria include no smoking, no caffeinated drinking, and no medications of hydrocortisone or prednisolone, etc. Other medications are allowed during the research. Saliva collections are non‐invasive and can be accepted by most human subjects. Therefore, patients under prescription and home care could collect their saliva samples and store in proper ways, and send these samples to the hospital for assessment of their circadian rhythms. We hope this objective measurement can precisely assess the circadian rhythms of these patients who may need future light therapy to treat their disorders. Although our results suggest that combined measurements of cortisol and melatonin can be considered as a biomarker to distinguish depressive BD patients and controls, our studies have limitations. First, the number of recruited subjects is relatively small. Second, we did not successfully recruit BD patients at the mania episode, which might provide more information if more mania patients are assessed. Though the number of recruited subjects is small, other studies support our findings that the phases of depressed patients are advanced than those in healthy controls (Gwirtsman et al., 1989). We would perform more studies to further understand the relationships between circadian clocks and different episodes in BD disease.

## CONFLICT OF INTEREST

The authors declare that they have no conflicts of interest.

## AUTHOR CONTRIBUTIONS

D. Z. and X.Q. conceived and designed research; J.Y. and Y. Z. collected the samples from recruited subjects; L.F., Q.Y., F.Y., J.Y, performed research; all authors were involved in analyzing data; L.F. wrote original draft, D.Z. and X.Q. writing ‐ review & editing.

### PEER REVIEW

The peer review history for this article is available at https://publons.com/publon/10.1002/brb3.2186.

## Supporting information

Figure S1‐S9
