# Supplementary material for: Combined cortisol and melatonin measurements with detailed parameter analysis can assess the circadian rhythms in bipolar disorder patients
Source: Brain Behav. 2021 Jun 6;11(7):e02186. doi: 10.1002/brb3.2186 (PMC8323050; doi:10.1002/brb3.2186)
Supplement: Supplementary file 1 — Figure S1‐S9 [file BRB3-11-e02186-s001.pdf]

Supplementary Figures

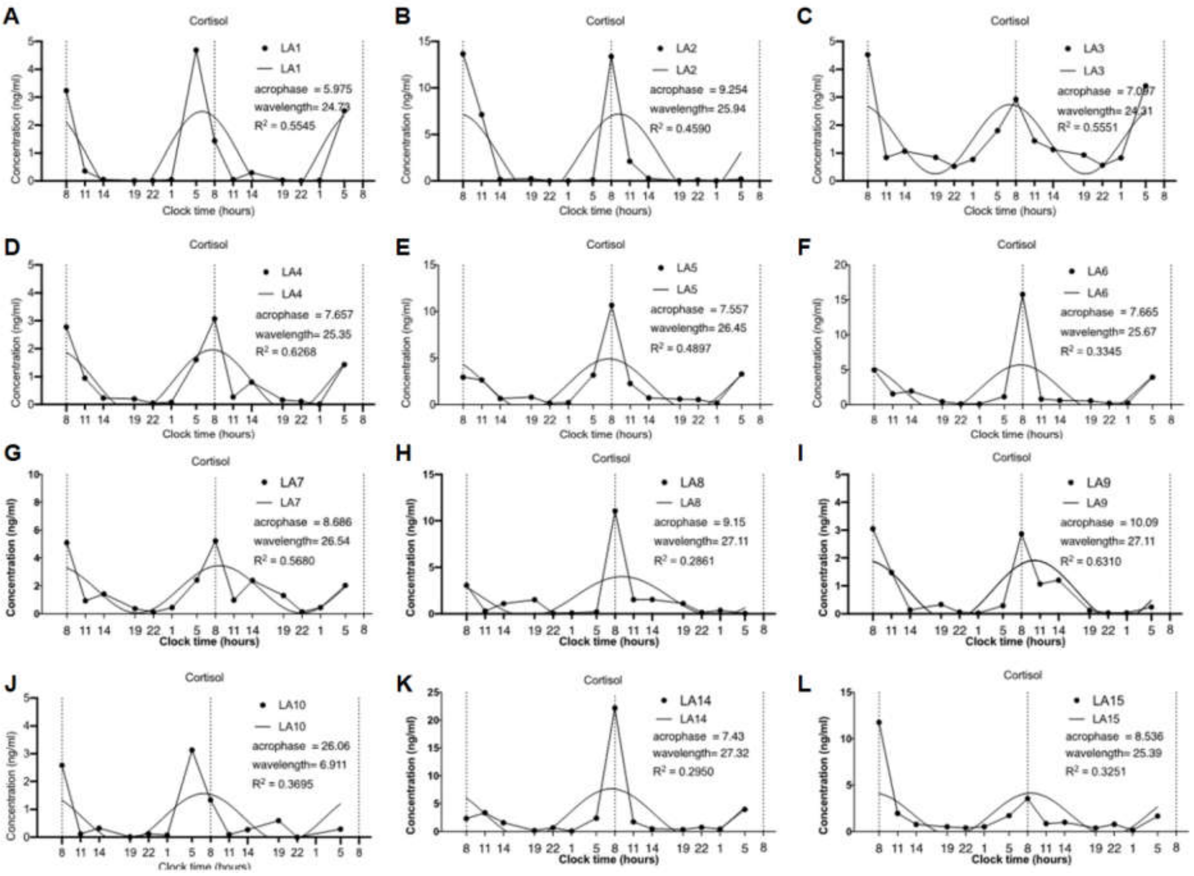

Figure S1.Cortisol profiles of individuals in healthy controls.

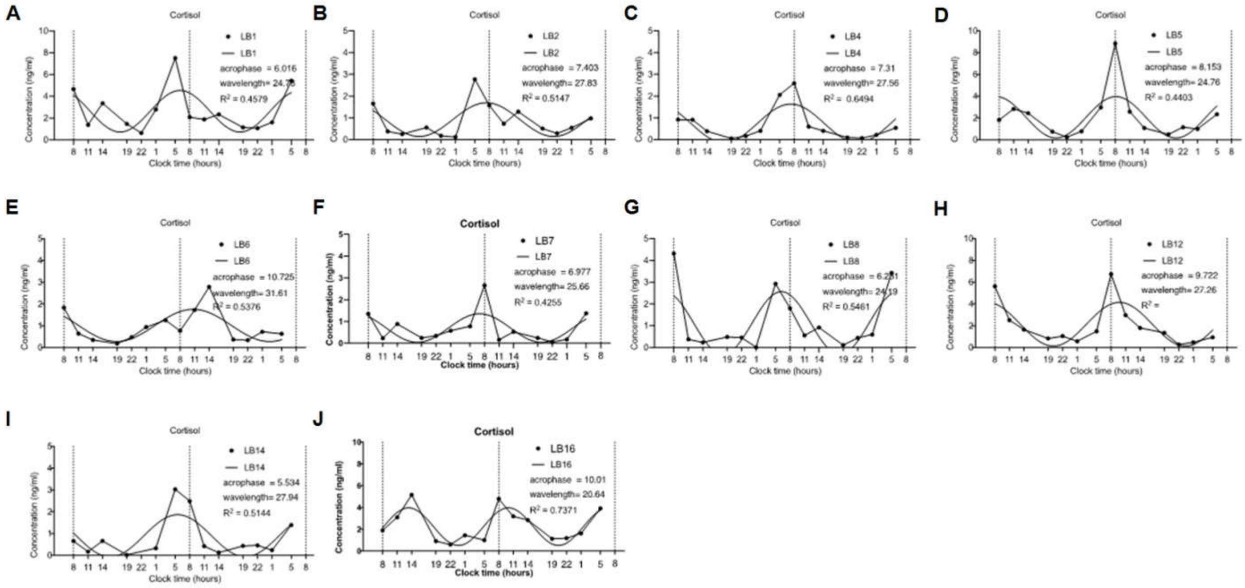

Figure S2. Cortisol profiles of individuals in BD patients at depression episode.

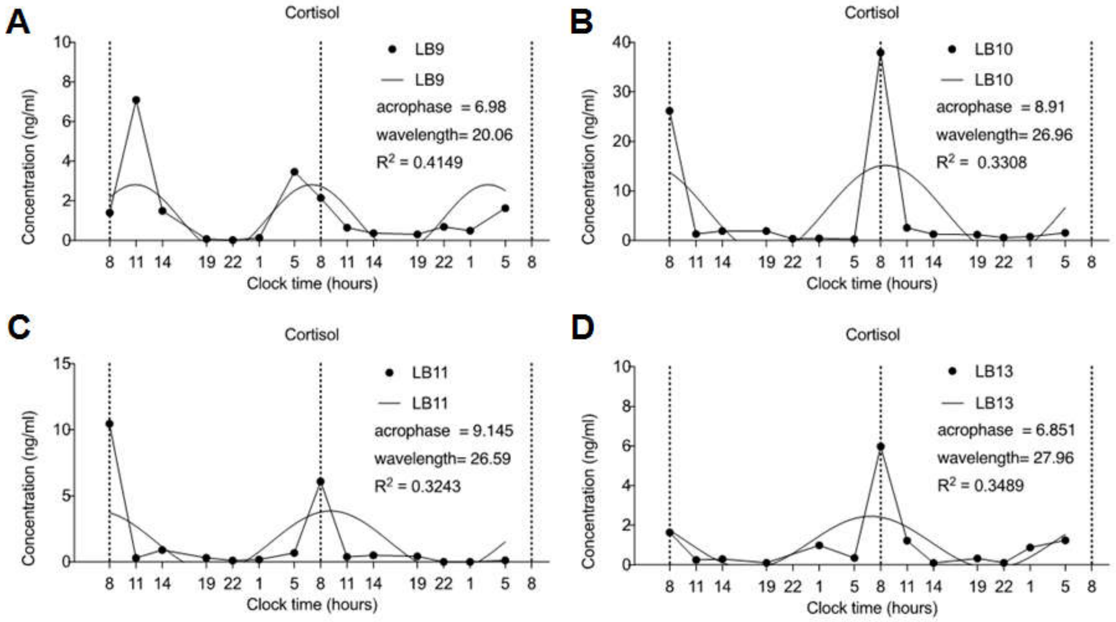

Figure S3. Cortisol profiles of individuals in BD patients at recovery stage.

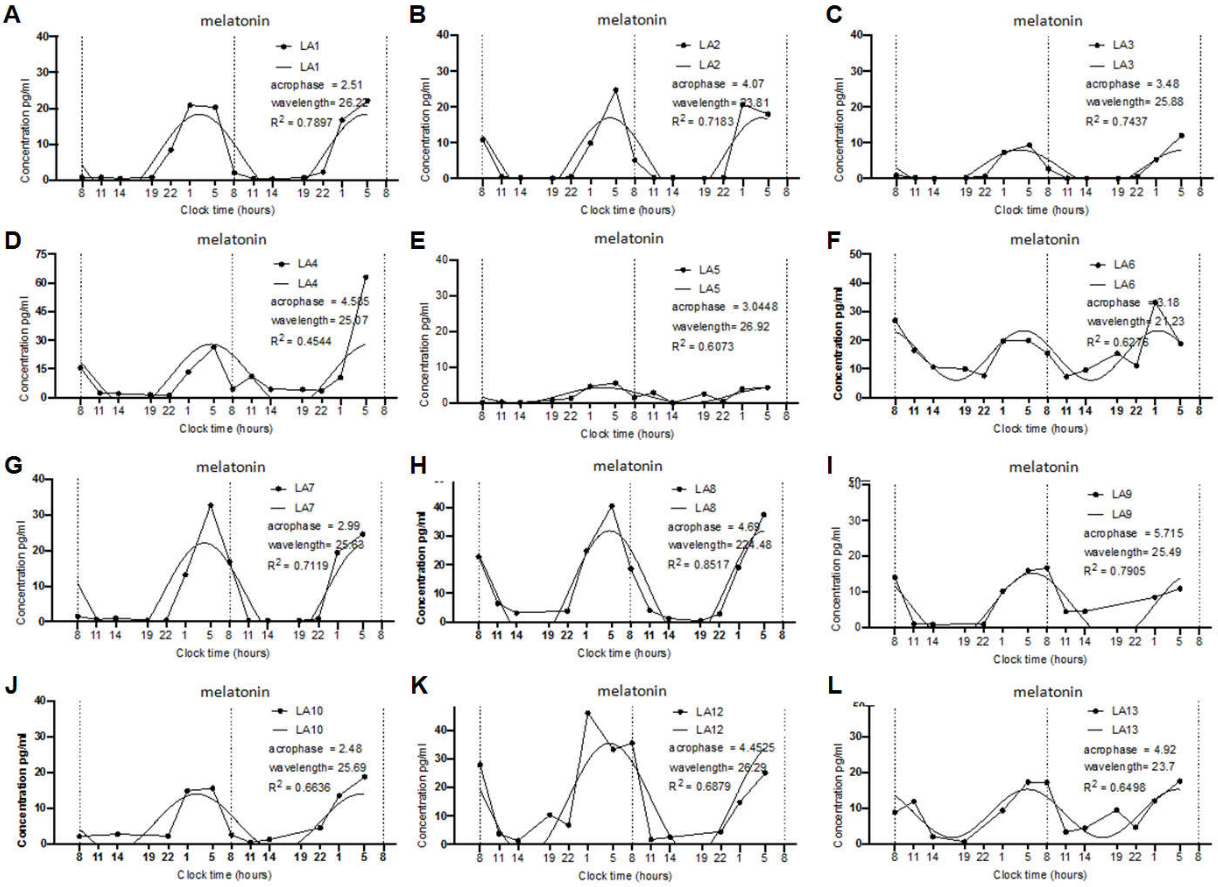

Figure S4. Melatonin profiles of individuals in healthy controls.

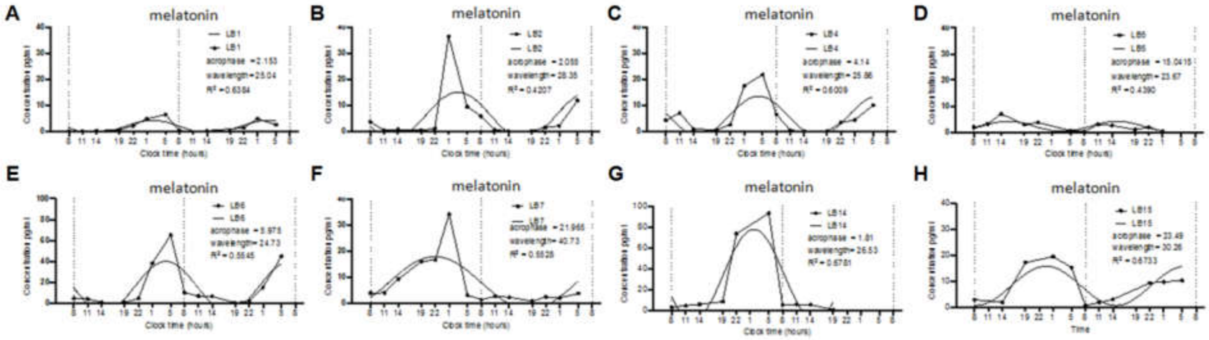

Figure S5. Melatonin profiles of individuals in BD patients at depression episode.

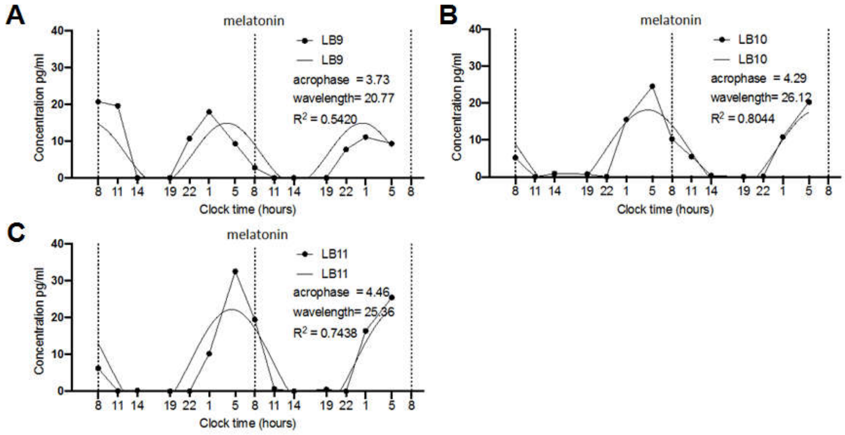

Figure S6. Melatonin profiles of individuals in BD patients at recovery stage.

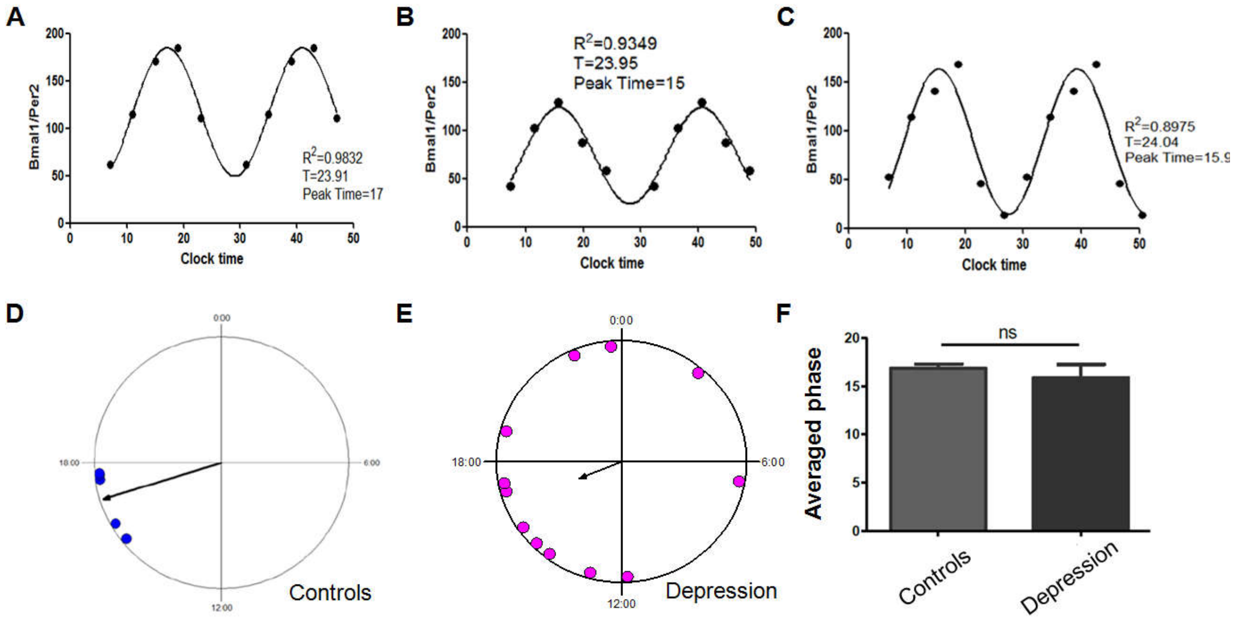

Figure S7. Gene expression profiles of the recruited subjects, which showed even distributions in both groups.

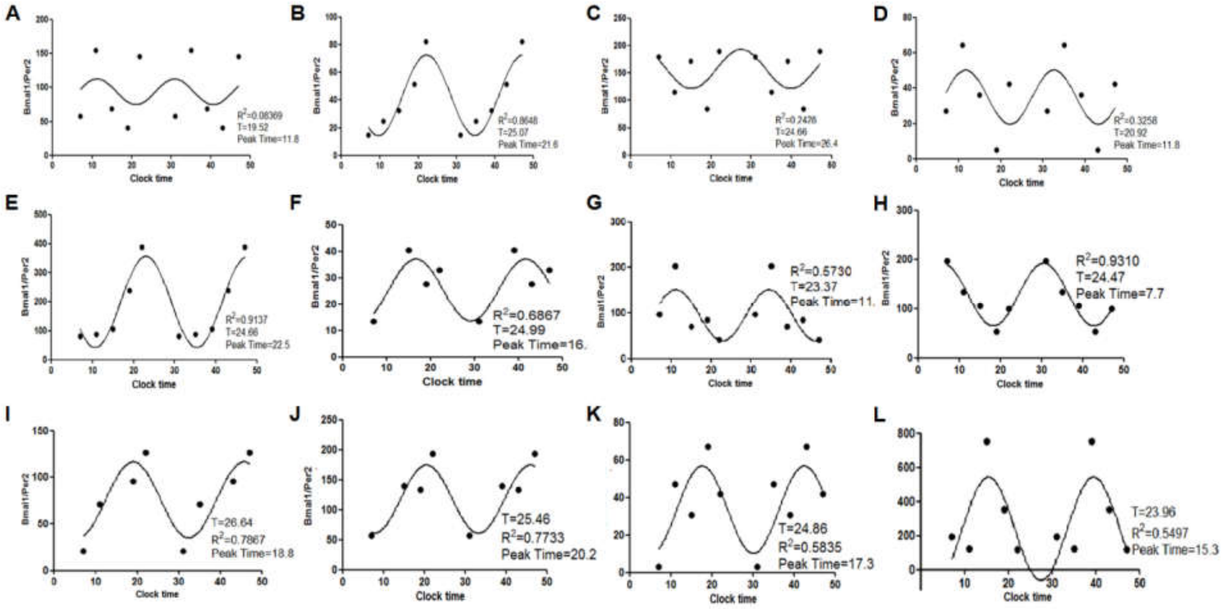

Figure S8. Gene expression profiles of individuals in BD patients at depression episode.

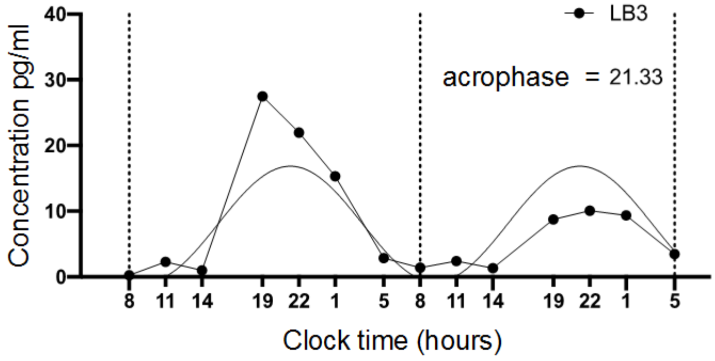

Figure S9. The melatonin profile of the BD patient at mania episode is highly phase advanced.
